# The Miraculous Cures of Lourdes

**Published:** 1892-10-01

**Authors:** R. F. Clarke


### Editor's Letter Box.

[Our correspondents are reminded that prolixity of statement is a great bar to publication, and that brevity of style and conciseness of statement greatly facilitate early insertion.]

### THE MIRACULOUS CURES OF LOURDES.

The Protestant Medical Testimony.

The Rev. R. F. Clarke, S.J., the author of several works relating to the miracles of Lourdes, having in his most recent publication, entitled "Medical Testimony to the Miracles of Lourdes," just issued by the Catholic Truth Society, referred in this connection to "the testimony of some three hundred medical men, among whom are many Protestants and unbelievers, who bear witness to a healing change in their patients, which no human agency known to science can explain," a correspondent wrote to him asking for a list of the "Protestants and unbelievers" who were thus stated to have given medical testimony to the reality of the miraculous cures at Lourdes. The following reply has been received:—

Church of the Holy Name, Manchester.
September 15th, 1892.

My Dear Sir,—I fear that it would be difficult, if not impossible, to obtain a list of the non-Catholic doctors who bear testimony to the miracles of Lourdes. Most of them are Frenchmen, practising in France, and would not like to be labelled as unbelievers. They come and visit Lourdes, and are present at the medical interrogatory of those who have been cured without any inquiry being made as to their beliefs, and it is only after they are gone that the resident physician learns that they are sceptics. Or they give the certificate of previous disease and subsequent sound health, and the fact of their being non-believers is known to the patient, but does not appear from their certificate. Dr. Charcot, the French hypnotist, sends many of his patients to Lourdes, and Dr. Bernheim says, "The facts of Lourdes now belong to science, only the explanation of them is in dispute." If it is possible to obtain for you any list of non-believers who have borne testimony to the miracles you shall have it.—I remain, Yours faithfully,

R. F. Clarke, S.J.

